# Phospholipase C in Beijing strains of *Mycobacterium tuberculosis*

**Published:** 2010-12

**Authors:** H Goudarzi, ES Mirsamadi, P Farnia, S Jahani Sherafat, M Esfahani, N Faramarzi

**Affiliations:** 1 Department of Microbiology, Faculty of Medicine, Shahid Beheshti University of Medical Sciences, Tehran, Iran; 2 Mycobacterium Research Center (MRC) National Research Institute of Tuberculosis and Lung Disease (NRITLD), Tehran, Iran; 3 School of Medicine, Shahid Beheshti University of Medical Sciences, Tehran, Iran

**Keywords:** *Mycobacterium tuberculosis*, Beijing strains, phospholipase C

## Abstract

**Background and Objectives:**

Phospholipase of *Mycobacterium tuberculosis* plays an important role in pathogenesis through breaking up phospholipids and production of diacylglycerol. In this study, we examined the Beijing strains of *Mycobacterium tuberculosis* isolated from Iranian patients for the genes encoding this enzyme.

**Materials and Methods:**

DNA extraction was performed using CTAB (cetyltrimethylammonium bromide) from positive culture specimens in tuberculosis patients. PCR was then used to amplify the *plcA, plcB, plcC* genes of Beijing strain, and non-Beijing strains were identified by spoligotyping.

**Results:**

Of 200 specimens, 19 (9.5%) were Beijing strain and 181 (90.5%) were non-Beijing strains. The results of PCR for Beijing strains were as follows: 16 strains (84.2%) were positive for *plcA*, 17 (89.4%) were positive for *plcB* and 17 (89.4%) were positive for *plcC* genes. The standard strain (H37RV) was used as control.

**Conclusion:**

The majority of Beijing strains have phospholipase C genes which can contribute to their pathogenesis but we need complementary studies to confirm the role of phospholipase C in pathogenecity of *Mycobacterium tuberculosis*.

## INTRODUCTION

*Mycobacterium tuberculosis* (TB) infects one-third of the world population (1). Although this organism can infect any area of the body including bones, joints, liver, spleen, gastrointestinal tract, and brain, it is primarily a pulmonary disease that is initiated by the deposition of *Mycobacterium tuberculosis* contained in aerosol droplets onto lung alveolar surfaces. With attention to the fact that *Mycobacterium tuberculosis* is still one of the biggest killers among the infectious diseases, worldwide use of a live attenuated vaccine and several antibiotics, it is essential to gain a better understanding of the pathogenicity and the virulence factors of *Mycobacterium tuberculosis* (2)**.

One of these virulence factors is phospholipase C. Phosphlipase C plays an important role in pathogenesis (3). Hydrolysis of phospholipids by Phospholipase C generates diacylglycerol that can act as an important signaling molecule in infected macrophages as well as a precursor for synthesis of triacylglycerol, which acts as an energy store for utilization during long- term dormancy of *M. tuberculosis* (3).

Phospholipase C genes include A, B, C and D segments. Three of these genes, *plcA*, *plcB* and *plcC*, are organized in tandem (locus *plcABC*). The fourth gene, *plcD*, is located in a different region (4). In addition to *Mycobacterium tuberculosis*, phospholipases are important virulence factors in an increasing number of intra- and extracellular bacterial pathogens including *Clostridium perfringens*, *Corynebacterium pseudotuberculosis*, *Pseudomonas aeruginosa*, *Staphylococcus aureus* and *Listeria monocytogenes*. Although bacterial PLCs are thought to be key virulence factors in several infectious diseases, the pathogenic mechanisms are quite varied. For example, clostridial -toxin is a PLC with haemolytic and lethal dermonecrotic and platelet-aggregating properties while that of *Listeria monocytogenes* functions to allow the organism to escape from intracellular phagolysosomes and purified PlcH preparations from *P. aeruginosa* are cytotoxic since injection into mice causes hepatonecrosis and renal tubular necrosis. This PlcH also inhibits the bacterium-induced neutrophil respiratory burst by interfering with a protein kinase C-dependent pathway (4, 5).

Tuberculosis remains a global threat to public health. The problem is further complicated by the emergence of multidrug-resistant tuberculosis as a consequence of the widespread use and incorrect administration of antibiotics (6). In these conditions, the Beijing MTB strain has attracted special attention because of its global emergence and resistance to multiple drugs (7, 8). For this reason we selected Beijing strains for our study. The aim of this study was the detection of phospholipase C genes in Beijing strains of *Mycobacterium tuberculosis* and relation between presence of phospholipase C and pathogenesis.

## MATERIALS AND METHODS

**Patients and samples**. This study involved a total of 200 patients that were referred to the National Research Institute of Tuberculosis and Lung Disease (NRILTD), the referral tuberculosis center in Iran, during August 2008 to August 2009.

All isolates were identified as *M. tuberculosis* by using biochemical tests, including production of niacin, catalase activity, nitrate reduction, pigment production and growth rate. Drug susceptibility testing against isoniazid (INH), rifampicin (RF), streptomycin (SM), and ethambutol (ETB) were performed by the proportional method on Löwenstein-Jensen media at a concentration of 0.2,40,10 and 2.0 µg/ml, respectively (9). The isolation of genomic DNA was carried out by the CTAB (cetyltrimethylammonium bromide) method (10).

**Spoligotyping**. All the isolates were studied based on spacer types as described previously by Kamerbeek et al. (11). In Spoligotyping, the DR region was amplified by PCR using primers derived from a DR sequence. The amplified DNA hybridised to a set of 43 immobilised oligonucleotides derived from the spacer sequences of *M. tuberculosis* H37RV and *M. bovis* BCG P3 by reverse line blotting (12). Spoligotyping is currently the gold standard for the identification and classification of Beijing strains of MTB (7).

**PCR of** ***plcabc***. In this study, PCR amplification of *plcA*, *plcB* and *plcC* genes was performed using previously published primers by Talarico, et al. (13). The sequences of gene-specific primers were as indicated in Table 1.

**Table 1:** Oligonucleotide primers used in different PCR assays.

designation	Sequence 5′	3′	⟶
*plcA*-PCR	plcA-F	5**′**TCG AAC GCC GGG AGA TTA CC 3**′**	
	plcA-R	5**′**GCA GGA AGG CAG GGC AAG TG3	
*plcB*-PCR	plcB-F	5**′**-TCC GGC GAA TGC ACC TTG GCT CAC-3**′**	
	plcB-R	5**′**-CGG CAG GCA GGC GGA ATC AGA ACA-3'	
*plcC*-PCR	plcC-F	5**′**-GGG CGG CAA AGG CGG ACC AAG AG-3**′**	
	plcC-R	5**′**-AAG CCG AAA TAC ACG AGG GAG AGC-3'	

**Data analysis:** Statistical analysis was done with SPSS (version 11.5). Chi-square and Fisher exact test were applied: P value<0.05 was considered significant.

The amplification reaction was performed in a 25 volume reaction mix containing 2.5 µl PCR buffer, 1 mM MgCl, 0.5 µm each of four dNTP, 2 µm each of primers, 0.15 U of Taq polymerase, 2 µl of extracted DNA from sample. PCR amplification was performed in a DNA thermal cycler set for initial denaturation run at 94°C for 1 min, denaturation at 94°C for 30 s, anealing at 62°C, 65°C, 67°C for 30 s in *plcA*, *plcB* and *plcC* genes, respectively, extension at 72°C for 150 s and final extension at 72°C for 10 min for 26 cycles. The amplification products, together with 100bp ladder fragments, were electrophoresed on ethidium bromide.

## RESULTS

**Fig. 1:**
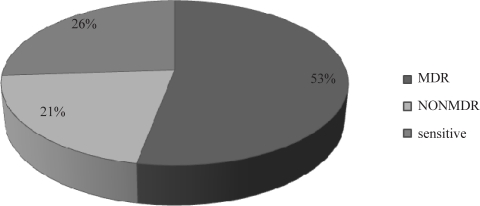
Result of antibiotic susceptibility tests for Beijing strains.

**Fig. 2:**
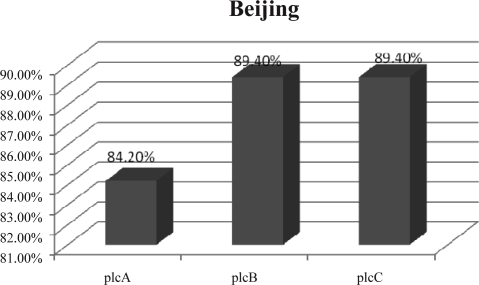
The frequency of various Phospholipase C genes in Beijing strain of *M. tuberculosis*.

Out of 200 specimens, 19 (9.5%) were Beijing strain and 181 (90.5%) were non Beijing strains using spoligotyping. In Drug susceptibility testing, MDR rate in Beijing strains was 53% (Fig. 1) and in nonbeijing strains was 10%. We used PCR method for detection of *plcA, plcB,and plcC* genes. In Beijing strains,16 specimens (84.2%) were positive for *plcA*, 17 (89.4%) were positive for *plcB* and 17 (89.4%) were positive for *plcC* genes (Fig. 2). The standard strain (H37RV) was used as control. The PCR amplification by *plcA*-F and *plcA*-R yielded a 450bp amplicon which corresponds to a conserved region of the *plcA* gene.The PCR amplification by *plcB*-F and *plcB*-R yielded a 1300bp amplicon which corresponds to a conserved region of *plcB* gene and the amplification by *plcC*-F and *plcC*-R yielded a 500bp amplicon which corresponds to a conserved region of *plcC* gene.

## DISCUSSION

Because of the demonstrated role of PLC in pathogenicity of an increasing number of pathogenic bacteria, including *Mycobacterium tuberculosis*, in the present study we detected phospholipase C genes in *M. tuberculosis* but we worked on Beijing strains because of the relation between these strains and multidrug-resistance (7). The primary aim of this study was: 1) determination of drug susceptibility in clinical isolates, 2) determination of spoligotyping pattern of the clinical strain isolated from patients,3) determination of the extent of Beijing strains prevalence among the clinical isolates, and 4) detection of the prevalence of *plcA*, *plcB*, and *plcC* genes by PCR amplification with specific primers among Beijing strains. As a result, we showed that the frequency of *plcA*, *plcB*, and *plcC* genes in Beijing strain were 84.2%, 89.4%, 89.4%, respectively that can pertain to Beijing strain pathogenesis.

Various studies have also suggested that phospholi- pases are found in a wide variety of Gram-positive and Gram-negative bacteria including *Clostridium perfringens*, *Corynebacterium pseudotuberculosis*, *Pseudomonas aeruginosa*, *Staphylococcus aureus* and *Listeria monocytogenes* and are generally categorized by substrate specificity. Some of these cleave the phospholipid phosphatidylcholine (PC) to produce phosphorylcholine and diacylglycerol (DAG) while some cleave other important phospholipid substrates such as phosphatidylinositol (PI) or sphingomyelin (SM). The production of DAG may be particularly important in pathogenesis. Indeed, phospholipase C by activating the arachidonic acid cascade may interfere with signal transduction events in infected cells, thus modulating the host immune responses (14–16).

In a similar study, Reverse transcription (RT)- PCR assays provided evidence that the *plc* genes are induced during the infection of human THP-1-derived macrophages and showed that phospholipase activities are higher in mycobacteria grown in mice than in those grown in the lipid-free Dubos medium (17).

Another investigation indicated that the expression of the *plc* genes of other pathogenic bacteria are also upregulated during host infection. These facts support the idea that the expression of the *M. tuberculosis plc* genes is upregulated in macrophages and phospholipase C plays a role in host infection. Consistent with this hypothesis, the disruption of the *plcABCD* or *plcABC* genes impaired the ability of *M. tuberculosis* to multiply in the lungs and spleen of infected mice. This is the first evidence that phospholipase C is required for the full virulence of *M. tuberculosis* (18, 19) **. Another study revealed a limited role for PlcD in the virulence of the tubercle bacillus. They showed that three out of seven clinical isolates analysed were deficient in expression of *plcD* (20).

## CONCLUSION

Phospholipase C genes can be important in pathogenesis of Beijing strains of *M. tuberculosis*, but confirmative studies need to be performed to verify the role of phospholipase C in pathogenecity of *Mycobacterium tuberculosis*.

## References

[1] Kam KM, Yip CW, Tse LW, Leug Kl, Wong KL, Ko WM (2006). Optimization of variable number tandem repeat typing set for diferentiating *Mycobacterium tuberculosis* in the Beijing family. FEMS Microbiol Lett.

[2] Issar S (2003). *Mycobacterium tuberculosis* pathogenesis and molecular determinants of virulence. J Clin Microbiol.

[3] Srinivas M, Rajakumari S, Narayana Y, Joshi B, Katoch VM, Rajasekharan R (2008). Functional characterization of the phospholipase C activity of Rv3487c and its localization on the cell wall of *Mycobacterium tuberculosis*. J Biosci.

[4] Vera- Cabrera L, Howard ST, Laszlo A, Johnson WM (1997). Analysis of genetic polymorphism in the phospholipase region of *Mycobacterium tuberculosis*. J Clin Microbiol.

[5] Ramazanzadeh R, Amirmozafari N, Farnia P, Ghazi F (2006). Genotyping of *Mycobacterium tuberculosis* isolate from TB patients with spoligotyping. J Kurdistan Uni Med Sci.

[6] Rohani M, Farnia P, Naderi Nasab M, Moniri R, Torfeh M, Amiri MM (2009). Beijing genotype and other predominant *Mycobacterium tuberculosis* spoligotypes observed in Mashhad. Indian J Med Microbiol.

[7] Sun JR, Lee SY, Dou HY, Lu JJ.Using, a multiplex K, Cole ST (2009). Identification of variable regions in polymerase chain reaction for the identification of Beijing strains of *Mycobacterium tuberculosis*. Eur J Clin Microbiol Infect Dis.

[8] Ferdinand S, Sola C, Verdol B, Legrand E, Goh KS, Berchel M (2003). Molecular characterization and drug resistance patterns of strains of *Mycobacterium tuberculosis* isolated from patients in an AIDS counseling center in Port-au-Prince, Haiti: a 1-year study. J Clin Microbiol.

[9] Schaaf HS, Shean Donald PR (2003). Culture confirmed multidrug resistant tuberculosis: diagnostic delay, clinical features, and outcome. Arch Dis Child.

[10] van Soolingen D, Hermans PW, de Haas PE, Soll DR, van Embden JD (1991). Occurrence and stability of insertion sequences in *Mycobacterium tuberculosis* complex strains: evaluation of an insertion sequence-dependent DNA polymorphism as a tool in the epidemiology of tuberculosis. J Clin Microbiol.

[11] Kamerbeek J, Schouts L, Kolk A, Agterveld V, van Solingen D, Kuijper S (1997). Simultaneous detection and strain differentiation of *Mycobacterium tuberculosis* for diagnosis and epidemiology. J Clin Microbiol.

[12] Farnia P, Mohammadi F, Masjedi MR, Varnerot A, Zarifi AZ, Tabatabee J (2004). Evaluation of tuberculosis transmissionin Tehran; using RFLP and spoligotyping. J Infect.

[13] Talarico S, Durmaz R, Yang Z (2004). Insertion and deletion- assoaiated genetic diversity of *Mycobacterium tuberculosis* phospholipase C- encoding gene among 106 clinical isolates from Turkey. J Clin Microb.

[14] Korbsrisate S, Tomaras P, Damnin S, Kumdee J, Srinon V, Lengwehasatit I (2007). Characterization of two distinct phospholipase C enzymes from *burkholderia pseudomallei*. J Clin Microbiol.

[15] Gomez A, Mve-Obiang A, Vray B, Rudnicka W, Shamputa I, Portaels F (2001). Detection of phospholipase C in nontuberculous mycobacteria and its possible role in hemolytic activity. J Clin Microbiol.

[16] Marquis H, Goldfine H, Daniel A, Portnoy DA (1997). Proteolytic pathways of activation and degrada-tion of a bacterial phospholipase C during intra-cellular infection by *Listeria monocytogenes*. J Cell Biol.

[17] Wheeler PR, Ratledge C (1992). Control and location of acyl-hydrolysing phospholipase activity in pathogenic mycobacteria. J Gen Microbiol.

[18] Agaisse H, Gominet M, okstad O.A, Kolsto A.B, Lereclus D (1999). PlcR is a pleiotropic regulator of extracellular virulence factor gene expression in *Bacillus thuringiensis*. J Mol Microbiol.

[19] Marquis H, Hager E.J (2000). pH-regulated activation and release of a bacteria-associated phospholipase C during intracellular infection by *Listeria mono-cytogenes*. J Mol Microbiol.

[20] Gordon SV, Brosch R, Billault A, Garnier T (1999). Eiglmeier the genomes of tubercle bacilli using bacterial artificial chromosome arrays. J Mol Microbiol.

